# Physical activity and sleep duration during pregnancy have interactive effects on caesarean delivery: a population-based cohort study in Tianjin, China

**DOI:** 10.1186/s12884-021-03788-4

**Published:** 2021-05-28

**Authors:** Yingzi Yang, Weiqin Li, Wen Yang, Leishen Wang, Jinnan Liu, Junhong Leng, Wei Li, Shuo Wang, Jing Li, Gang Hu, Zhijie Yu, Xilin Yang

**Affiliations:** 1 Department of Epidemiology and Biostatistics, School of Public Health, Tianjin Medical University, P.O. Box 154, 22 Qixiangtai Road, Heping District, Tianjin 300070, China; 2 Project Office, Tianjin Women and Children’s Health Center, Tianjin, China; 3 Department of Child Health, Tianjin Women and Children’s Health Center, Tianjin, China; 4 Chronic Disease Epidemiology Laboratory, Pennington Biomedical Research Center, Baton Rouge, Louisiana, USA; 5 Population Cancer Research Program and Department of Pediatrics, Dalhousie University, Halifax, Canada; 6 Tianjin Key Laboratory of Environment, Nutrition and Public Health, Tianjin, China; 7 Tianjin Center for International Collaborative Research on Environment, Nutrition and Public Health, Tianjin, China

**Keywords:** Physical activity, Sleep duration, Caesarean delivery, Pregnancy, Chinese

## Abstract

**Background:**

There were inconsistent findings in the literature regarding the associations of physical activity and sleep duration during pregnancy with caesarean delivery for different reasons. It was also unknown whether physical activity and sleep duration during pregnancy had interactive effects on the risks of different types of caesarean delivery. The study aimed to investigate the effects of physical activity, sleep duration and their interactions on the risk of caesarean delivery for medical reasons and non-medical reasons.

**Methods:**

From October 2010 to August 2012, a prospective population-based cohort of 13,015 pregnant women was established in six central urban districts of Tianjin, China. Pregnancy outcomes were retrieved from an electronic database and caesarean delivery was divided into caesarean delivery for medical reasons and caesarean delivery for non-medical reasons. Physical activity and sleep status were collected at 24–28 weeks of gestation using self-reported questionnaires. Logistic regression and additive interaction were used to examine physical activity, sleep duration and their interactive effects on risk of caesarean delivery.

**Results:**

In the cohort, 5692 (43.7%) and 2641 (20.3%) of women had caesarean delivery for medical reasons and non-medical reasons, respectively. Low physical activity increased the risk of caesarean delivery for medical reasons (adjusted OR: 1.13, 95%CI 1.04–1.23) but not caesarean delivery for non-medical reasons. Sleep duration < 7 h/day and poor sleep quality were not associated with caesarean delivery. Sleep duration ≥9 h/day increased the risk of caesarean delivery for medical reasons (1.12, 1.02–1.22) and caesarean delivery for non-medical reasons (1.16, 1.05–1.29). Co-presence of low physical activity and sleep duration ≥9 h/day increased risk of caesarean delivery (1.25, 1.12–1.41), and their additive interaction was statistically significant for caesarean delivery for medical reasons but not for caesarean delivery for non-medical reasons.

**Conclusions:**

Low physical activity and excessive sleep duration during pregnancy each increased the risk of caesarean delivery, and they had an interactive effect on the risk of caesarean delivery for medical reasons but not on the risk of caesarean delivery for non-medical reasons. Increasing physical activity and maintaining recommended sleep duration during pregnancy may have benefits for perinatal health.

**Supplementary Information:**

The online version contains supplementary material available at 10.1186/s12884-021-03788-4.

## Background

Globally, caesarean delivery (CD) rate had doubled to 21.1% in the past 15 years and increased annually by 4% [1, 2]. China had the highest rate of CD in the world and the rate was also on the rise in the past decades, increased from 46.2% in 2004 to 47.6% in 2011 [3]. The underlying reasons were multifaceted, including policy, clinical decisions, socioeconomic status and psychological state of pregnant women [4, 5]. CD was performed with or without medical indications in clinical practice and had short-term and long-term health consequences such as maternal mortality and neonatal physiology alteration, especially when carried out without medical indications [6]. Thus, curbing the undue increase of CD rate in China has become a critical problem that needs to be solved [7].

Physical activity (PA) was a well-established factor for pregnancy complications. High-level PA was associated with decreased risk of gestational diabetes mellitus (GDM) [8] and low-level PA was associated with increased risk of preterm delivery [9]. Maternal exercise intervention also decreased risk of gestational hypertension and macrosomia [10]. These pregnancy complications could increase the risk of CD, suggesting that PA was likely to associate with CD. A prospective cohort study found that women with low PA levels had higher rates of elective CD and emergency CD [11]. While two meta-analyses of randomized controlled trials failed to generate consistent findings regarding the effects of PA on CD; one found that planned aerobic exercise resulted in a significantly lower incidence of CD [12] while the other found that prenatal exercise had no effects on CD [13]. The effects of PA on CD remained inconclusive.

For adults aged 18–64 years, it’s recommended to have 7 to 9 hours’ sleep per night by the American National Sleep Foundation [14]. It’s established that short and/or long sleep duration was associated with placental abruption [15], GDM [16], preeclampsia and hypertension [17], and these medical conditions increase CD risk. Additionally, several studies found that less than 6 h sleep per day increased CD risk [18, 19] while a small cohort study with 200 subjects found that 8 and more hours of sleep per day was associated with a higher rate of assisted deliveries (CD and instrumental delivery) [20]. Indeed, the association between sleep duration and CD was less consistent and evidence for excessive sleep duration was insufficient. In addition, Sarah-Jane Paine [21] and Run Li [22] separately reported poor sleep quality increased risk of CD, while an observational study of sleep disturbances failed to find that poor sleep quality was associated with increased risk of CD [23]. Therefore, we also aimed to address the association between sleep quality and CD. In a previous analysis, our group reported that longer sleep duration and low PA increased the risk of GDM [24, 25], which was likely to contribute to a high CD risk. To our knowledge, there were no studies to explore the interactive effects of PA and sleep duration on the risk of CD athough PA was associated with sleep duration [26, 27].

Based on a large population-based prospective cohort of pregnant women in Tianjin, China, the current analyses aimed to explore (1) whether PA and sleep status during pregnancy were associated with CD risk; and (2) whether PA and sleep duration had a synergistic effect on the risk of CD in Chinese pregnant women.

## Methods

### Study population and settings

**Fig. 1:**
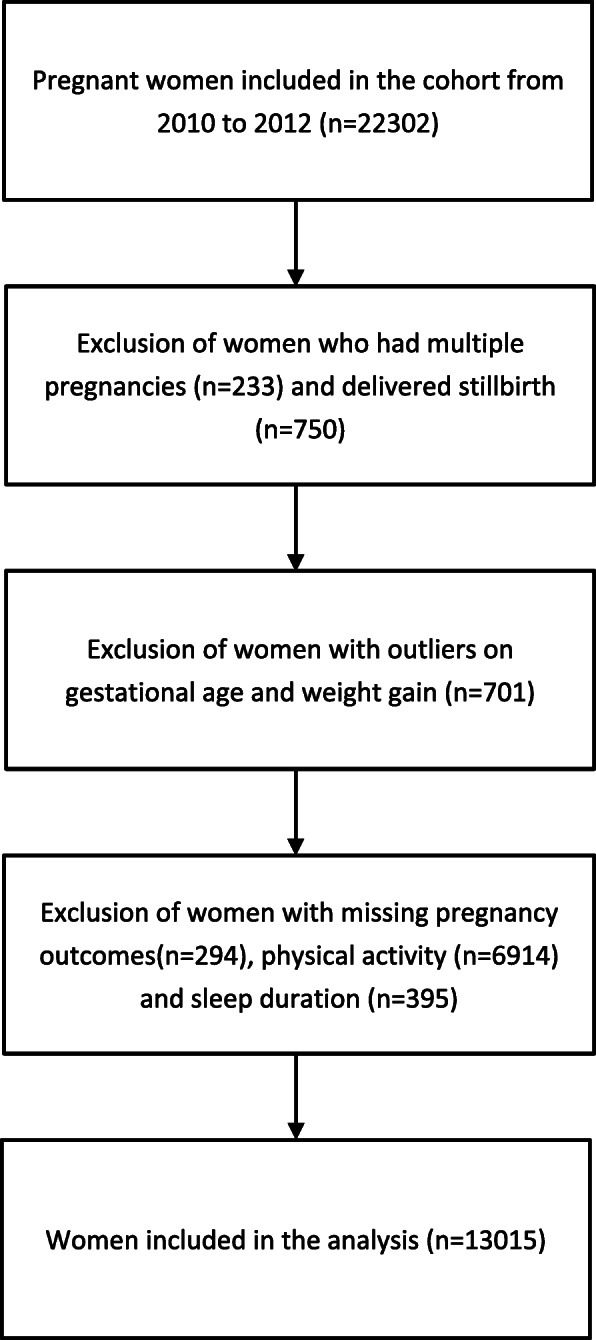
Study flow diagram of all the exclusions in the cohort

Using an established three-tiered screening and management system for GDM in 1999 in six central urban districts of Tianjin, China [28], our group set up a large population-based cohort of pregnant women and their offspring from October 2010 to August 2012 [29]. A total of 22,302 pregnant women made their first antenatal care visit to primary care hospitals close to their residence during the study period. Among them, we sequentially excluded 233 women with multiple pregnancies, 750 women who delivered stillbirth, 294 women with missing pregnancy outcomes, 701 women with outliers on gestational age and weight gain, 7309 women with missing PA (*n* = 6914) and sleep duration (*n* = 395). Eventually, 13,015 subjects were included in the analyses (Fig. 1).

### Clinical measurements and data collection of potential confounders

Clinical and biochemical data were collected longitudinally from their first antenatal care visit to delivery by a series of questionnaires or retrieved from the database of the Maternal and Child Health Information System [30]. Information on height, weight, body mass index (BMI), gestational age, systolic/diastolic blood pressure (SBP/DBP), weight at registration and glucose challenge test (GCT), maternal age, nationality, parity, family history of diabetes in first degree relatives, smoking and drinking habits were measured or collected by care providers. Weight gain was computed as the difference in weight between at the first antenatal care visit and at GCT time. GDM was identified using a two-step procedure: a 50-g 1-h GCT followed by a 75-g 2-h oral glucose tolerance test if the GCT was 7.8 mmol/L and more [31]. The International Association of Diabetes and Pregnancy Study Group (IADPSG) criteria were used to diagnose GDM [32].

### Assessment of physical activity and sleep status

PA was collected at the GCT time by a self-reported questionnaire. The questionnaire was used in a study [33] and also used in one of previous analyses of the cohort [25]. The detailed description of the methods and validation information were available elsewhere [34]. To collect data on PA accurately and comprehensively, we further collected information about housework PA. Briefly, occupational, commuting, leisure-time and housework physical activities were documented. Occupational PA was divided into three categories: low (unemployment or work always sitting indoor, e.g. desk work of a secretary), moderate (work with standing, walking or lifting, e.g. waiter), and high (work with heavy manual labor, e.g. industrial workers or athletes). Commuting PA was classified into two categories: low (barely outgoing, using motorized transportation or < 30 min/day walking or cycling) and moderate-to-high (≥30 min/day walking or cycling). Leisure-time PA was divided into two categories: no leisure-time PA or exercise less than 150 min per week (< 150 min/week) and exercise more than 150 min per week (≥150 min/week). Housework PA was divided into ≤1 h/day and > 1 h/day. To gain a representative indicator of PA, we regrouped PA into two categories based on the four types of activities above: low (low occupational and commuting PA, leisure-time PA < 150 min/week and housework PA ≤1 h/day), and moderate-to-high (any of the four categories: moderate and high levels of occupational PA, moderate-to-high commuting PA, leisure-time PA ≥150 min/week and housework PA > 1 h/day).

Sleep items were collected in the same questionnaire at the GCT time. For use in busy clinical settings in this study, we adapted and further modified two questions from a well-validated scale (The Pittsburg Sleep Quality Index, PSQI) for measurement of self-reported sleep duration and quality of sleep [35]. The questions to collect sleep status were ‘How many hours did you sleep every day during pregnancy including both day and night time?’ and ‘How did you feel about your sleep quality during pregnancy: good, moderate or poor?’. To be consistent with the classification of sleep duration in our previous investigation [24], we defined sleep duration into three categories: < 7 h/day, ≥7- < 9 h/day and ≥ 9 h/day. Sleep quality was classified into three categories: good, moderate and poor.

### Definition of caesarean delivery

CD status and related information were retrieved from the electronic database of the Maternal and Children’s Health Information System. CD was divided into two types, CD for medical reasons and CD for non-medical reasons. Medical indications of CD included fetal distress, cephalopelvic disproportion, scar uterus, abnormal fetal position (breech, transverse, etc.), placenta previa, umbilical cord prolapse, placental abruption, gestational severe complications (GDM, gestational hypertensive or other internal diseases), macrosomia, uterine malformation, uterine inertia, hysteromyoma, pathological pelvis, prolonged labor and cervical lesions. Non-medical indications included social factors, family factors and personal factors. CD without any reasons provided was also classified as the CD for non-medical reasons.

### Statistical analyses

R software (R version 4.0.0) was used to perform statistical analyses unless specified. A *P*-value of less than 0.05 for the two-tailed test was considered to be statistically significant. Normal distribution of continuous variables was test with Q-Q plots. Continusous variables were presented as mean ± SD or median (25th percentile, 75th percentile). Analysis of variance (ANOVA) (if the normal distribution and Levene test were not rejected) or the Kruskal-Wallis test where appropriate was used for comparisons of continuous variables among vaginal delivery, CD for medical reasons and CD for non-medical reasons. Bonferroni test or Kruskal-Wallis H test where appropriate was used to perform multiple comparisons. Categorical variables were presented as number (percentages). Chi-squared test or Fisher’s exact test where appropriate was used to compare categorical variables among the three groups.

Binary logistic regression was used to obtain the odds ratio (OR) and 95% confidence interval (CI). At first, we obtained ORs and 95% CIs of PA and sleep status for CD in univariable analyses (model 1). Then, we adjusted for potential confounding factors (model 2), including maternal age (continuous), BMI (continuous), weight gain from registration to GCT (continuous), gestational age at delivery (continuous), habitual smokers before and during pregnancy, alcohol drinkers before and during pregnancy, family history of diabetes, parity, ethnicity, SBP (continuous), birth weight (continuous) and neonatal height (continuous). Those confounding factors were selected for being statistically siginifanct different between the CD group and the non-CD group or considering having clinical significance for CD by researchers. Because PA and sleep status were risk factors of GDM, we further adjusted for GDM status to check whether GDM played an intermediate role in the associations of PA and sleep status with CD (model 3).

Subgroup analyses were carried out to examine the consistency in the effects of low PA on CD among different sleep duration groups, and the effects of excessive sleep duration on CD among different PA groups. Short sleep duration was not further analyzed because it was not significant in univariate and multivariate analyses. Synergistic effects between PA and sleep duration on CD risk were checked using additive interaction. Three indicators were used to evaluate additive interaction: relative excess risk due to interaction (RERI), attributable proportion due to interaction (AP) or synergy index (S). Relative excess risk due to interaction > 0, attributable proportion due to interaction > 0 or synergy index > 1 was regarded as significant additive interaction [36].

To further check different effects of PA, sleep status and their interactive effects on CD for medical reasons and non-medical reasons, we further performed multinomial logistic regression with two response levels, CD for medical reasons and CD for non-medical reasons using the Statistical Analysis System (SAS) release 9.3 (SAS Institute Inc., Cary, NC, USA).

Additional analyses were perfomed to check potential bias in the main analyses. First, we compared the clinical and biochemical characteristics of women excluded and those included in the analyses. Second, we re-included 979 women who had multiple pregnancies or delivered stillbirth to check the impacts of exclusion of those women on the main findings. Third, we analyzed the effect of PA classes, i.e., occupational, commuting, leisure-time and housework physical activities, on the risk of CD for medical reasons and CD for non-medical reasons to further explore the specific effect of PA classes.

## Results

### Characteristics of the study population

The mean age of 13,015 pregnant women was 28.5 (standard deviation, SD: 2.9) years, with 96.3% of them being primipara. The mean gestation age at registration was 10 (SD: 2.3) weeks. The mean pre-pregnancy BMI was 22.3 (SD: 3.4) kg/m^2^ and they delivered an infant at a means of 39.1 (SD: 1.5) weeks of gestation. A total of 8333 women (64.0%) had CD. The CD rates for medical reasons and non-medical reasons were 43.7 and 20.3%, respectively. The main medical indications of CD were cephalopelvic disproportion (32.8%), fetal distress (18.7%), others (18.0%), social factors (5.8%), and breech presentation (5.0%). Women with CD for medical reasons or non-medical reasons were older, had higher pre-pregnancy BMI, SBP and DBP, and more likely to smoke and have a family history of diabetes than women without CD. Infants born by CD had higher weight and height. Women with CD were more likely to have low PA and excessive sleep duration. Individually, women with CD for medical reasons or non-medical reasons had lower levels of occupational PA, commuting PA, leisure-time PA, housework PA, and longer sleep duration, while sleep quality were similar among the three groups (Table 1).

**Table 1:** Clinical and biochemical characteristics of subjects by three different delivery types

Variables	Vaginal delivery	CD for medical reasons	CD for non-medical reasons	*P*-value
n	4682	5692	2641	
**Variables at registration for pregnancy**				
Age, year	28.07 ± 2.60^†,‡^	28.72 ± 3.04^†^	28.66 ± 3.11^‡^	< 0.001^*^
Pre-pregnancy BMI, kg/m^2^	21.56 ± 2.97^†,‡^	22.81 ± 3.54^†^	22.62 ± 3.48^‡^	< 0.001^*^
Han-nationality	4481 (95.7%)	5404 (94.9%)	2516 (95.3%)	0.186^**^
Weight, kg	57.82 ± 8.68^†,‡^	60.67 ± 10.20^†,§^	60.09 ± 10.04^‡,§^	< 0.001^*^
Height, cm	163.68 ± 4.55^†,‡^	162.99 ± 4.82^†^	162.88 ± 4.74^‡^	< 0.001^*^
Systolic blood pressure, mmHg	104.80 ± 10.38^†,‡^	106.24 ± 10.82^†^	106.22 ± 11.10^‡^	< 0.001^*^
Diastolic blood pressure, mmHg	67.73 ± 7.50^†,‡^	68.95 ± 7.83^†^	69.00 ± 8.05^‡^	< 0.001^*^
Parity ≥1	138 (2.9%)	249 (4.4%)	97 (3.7%)	0.001^**^
Family history of diabetes in first degree relatives	364 (8.3%)	538 (10.0%)	223(9.1%)	0.014^**^
Gestational age at registration, weeks	10.45 ± 2.40^†^	10.32 ± 2.31^†^	10.39 ± 2.27	0.008^*^
History of caesarean delivery ≥1	5 (0.1%)	193 (3.4%)	64 (2.4%)	< 0.001^**^
**Variables at Glucose challenge test**				
Smoke before or during pregnancy	119 (2.5%)	326 (5.7%)	162 (6.1%)	< 0.001^**^
Drink before or during pregnancy	1462 (31.2%)	1818 (31.9%)	846 (32.0%)	0.680^**^
Gestational diabetes mellitus	261 (5.9%)	453(8.4%)	183 (7.4%)	< 0.001^**^
Glucose challenge test, mmol/L	6.49 ± 1.50^†,‡^	6.72 ± 1.60^†^	6.71 ± 1.56^‡^	< 0.001^*^
Weight gain from registration to GCT, kg	7.41 ± 3.02^†,‡^	7.81 ± 3.58^†^	7.72 ± 3.18^‡^	< 0.001^*^
Occupational physical activity				0.347^**^
Low	4022 (86.0%)	4946 (86.9%)	2289 (86.7%)	
Moderate-to-high	656 (14.0%)	743 (13.1%)	351 (13.3%)	
Commuting physical activity(min/day) ^a^				0.003^**^
low	4374 (93.6%)	5386 (94.7%)	2513 (95.4%)	
Moderate-to-high	301 (6.4%)	302 (5.3%)	122(4.6%)	
Leisure-time physical activity(min/week)				0.123^**^
0	2477(53.1%)	3074(54.3%)	1474(56.0%)	
0–150	1391 (29.8%)	1657 (29.2%)	716 (27.2%)	
≥ 150	797 (17.1%)	935 (16.5%)	441 (16.8%)	
Housework physical activity(h/day)				0.007^**^
≤ 1	3544 (77.6%)	4432 (79.9%)	2008 (77.6%)	
> 1	1022 (22.4%)	1114 (20.1%)	579(22.4%)	
Physical activity during pregnancy ^b^				
Low	2454 (52.4%)	3147(55.3%)	1427 (54.0%)	0.014^**^
Moderate-to-high	2228 (47.6%)	2545 (44.7%)	1214 (46.0%)	
Sleep duration during pregnancy(h/day)				0.025^**^
< 7	88 (1.9%)	98 (1.7%)	65(2.5%)	
≥ 7 to < 9	2067 (44.1%)	2410 (42.3%)	1091 (41.3%)	
≥ 9	2527 (54.0%)	3184 (55.9%)	1485 (56.2%)	
Sleep quality during pregnancy				0.872^**^
Good	1762 (37.7%)	2161 (38.1%)	990 (37.6%)	
Moderate	2792 (59.8%)	3390 (59.8%)	1583 (60.1%)	
Poor	114 (2.4%)	122(2.2%)	63 (2.4%)	
**Variables at delivery**				
Gestational age at delivery, weeks	39.09 ± 1.60^†^	39.06 ± 1.40^†,§^	39.13 ± 1.52^§^	< 0.001^*^
Birth weight, kg	3.28 ± 0.42^†,‡^	3.46 ± 0.49^†,§^	3.40 ± 0.45^‡,§^	< 0.001^*^
Neonatal height, cm	50.03 ± 1.67^†,‡^	50.21 ± 1.63^†^	50.22 ± 1.60^‡^	< 0.001^*^

*Abbreviations:* *BMI* body mass index; *GCT* glucose challenge test; *CD* caesarean delivery

^*^Derived from analyses of variance (ANOVA) or the Kruskal-Wallis test;

^**^Derived from Chi-square Test or Fisher’s exact test

For analyses of continuous variables, Bonferroni test or Kruskal-Wallis H test was used to perform multiple comparisons with identical †, ‡, or § indicating statistically significant differences between two means

^a^ Low was defined was defined barely outgoing, using motorized transportation or < 30 min/day walking or cycling to and from work

^b^ Low was defined when subjects simultaneously reported the light level of occupational physical activity, low commuting physical activity, < 150 min/week of leisure-time physical activity, and ≤ 1 h/day housework physical activity; and others were defined as ‘moderate-to-high’

### Physical activity and sleep status for caesarean delivery risk

Using moderate-to-high PA as the reference, the unadjusted OR and adjusted OR of low PA for CD were 1.10 (95%CI: 1.03–1.19) and 1.12 (95%CI: 1.03–1.21), respectively. Compared to sleep duration from ≥7 to < 9 h/day, sleep duration < 7 h/day was not associated with CD risk while sleep duration ≥9 h/day was significantly associated with increased risks of CD (unadjusted OR: 1.09, 95%CI: 1.01–1.17 & adjusted OR: 1.13, 95%CI: 1.04–1.22). Using good sleep quality as the reference, moderate and poor sleep quality were not associated with CD risk. Further adjustment for GDM status did not significantly attenuate the effect size of PA and sleep duration for CD (Table 2).

**Table 2:** ORs of physical activity and sleep status during pregnancy for caesarean delivery

Variables	n (%)	OR (95% CI)		
		Model 1	Model 2	Model 3
Physical activity				
Low	4574 (35.1%)	1.10 (1.03–1.19)	1.12 (1.03–1.21)	1.12 (1.04–1.22)
Moderate-to-high	3759 (28.9%)	1.00 (Reference)	1.00 (Reference)	1.00 (Reference)
Sleep duration (h/day)				
< 7	163 (1.3%)	1.09 (0.84–1.43)	1.05 (0.79–1.41)	1.04 (0.78–1.39)
≥ 7 to < 9	3501 (26.9%)	1.00 (Reference)	1.00 (Reference)	1.00 (Reference)
≥ 9	4669 (35.9%)	1.09 (1.01–1.17)	1.13 (1.04–1.22)	1.13 (1.04–1.22)
Sleep quality				
Good	3151 (24.2%)	1.00 (Reference)	1.00 (Reference)	1.00 (Reference)
Moderate	4973 (38.2%)	1.00 (0.92–1.07)	0.99 (0.91–1.07)	1.00 (0.92–1.09)
Poor	185 (1.4%)	0.91 (0.71–1.16)	0.88 (0.68–1.14)	0.91 (0.70–1.19)

*Abbreviations:* *n%* number of cases (% of number at risk); *OR* odds ratio; *CI* confidence interval

Model 1: Univariable analyses

Model 2: Multivariable analyses, adjusted for age, body mass index, weight gain from registration to glucose challenge test, gestational age at delivery, habitual smokers before and during pregnancy, alcohol drinkers before and during pregnancy, family history of diabetes in first-degree relatives, parity≥1, Han nationality, systolic blood pressure at registration for pregnancy, birth weight, neonatal height

Model 3: Further adjusted for gestational diabetes, in addition to the variables listed in model 2

### Additive interaction between physical activity and sleep duration for caesarean delivery

Compared to moderate-to-high PA, low PA was not associated with CD risk among women with sleep duration < 9 h/day. However, the unadjusted and adjusted ORs of low PA were significant among women with sleep duration ≥9 h/day (unadjusted OR: 1.16, 95%CI: 1.06–1.28 & adjusted OR: 1.20, 95%CI: 1.08–1.34).

Among women with low PA, the unadjusted and adjusted ORs of sleep duration ≥9 h/day for CD were 1.15 (95%CI:1.04–1.27) and 1.21 (95%CI:1.09–1.35), respectively. Among women with moderate-to-high PA, sleep duration ≥9 h/day was not associated with the risk of CD.

Using the moderate-to-high PA and sleep duration < 9 h/day as the reference, presence of low PA alone and sleep time ≥ 9 h/day alone were not associated with increased risk of CD. However, co-presence of low PA and sleep duration ≥9 h/day significantly increased the risk of CD (adjusted OR: 1.25, 95%CI: 1.12–1.41) with significant additive interaction (Relative excess risk due to interaction: 0.17, 95%CI: 0.01–0.34; Attributable proportion due to interaction: 0.14, 95%CI: 0.01–0.27). The additive interaction between low PA and sleep duration ≥9 h/day for CD persisted after adjustment for GDM (Table 3).

**Table 3:** Subgroup and additive interaction analyses of physical activity and sleep duration for CD

Variables	OR (95% CI)		
	Model 1	Model 2	Model 3
**Subgroup analyses**			
Among sleep time < 9 h/day			
Low V.S Moderate-to-high PA	1.04 (0.94–1.16)	1.04 (0.92–1.17)	1.03 (0.91–1.16)
Among sleep time ≥ 9 h/day			
Low V.S Moderate-to-high PA	1.16 (1.06–1.28)	1.20 (1.08–1.34)	1.22 (1.09–1.36)
Among low PA			
≥9 h/day V.S < 9 h/day sleep time	1.15 (1.04–1.27)	1.21 (1.09–1.35)	1.22 (1.10–1.37)
Among moderate-to-high PA			
≥9 h/day V.S < 9 h/day sleep time	1.03 (0.92–1.14)	1.03 (0.92–1.16)	1.02 (0.90–1.15)
**Additive interaction models**			
Moderate-to-high PA and sleep time < 9 h/day	1.00 (Reference)	1.00 (Reference)	1.00 (Reference)
Low PA and sleep time < 9 h/day	1.04 (0.94–1.16)	1.04 (0.92–1.16)	1.03 (0.91–1.16)
Moderate-to-high PA and sleep time ≥ 9/day	1.03 (0.92–1.14)	1.04 (0.93–1.17)	1.03 (0.92–1.16)
Low PA and sleep time ≥ 9 h/day	1.20 (1.08–1.33)	1.25 (1.12–1.41)	1.25 (1.12–1.41)
**Additive interaction indications**			
RERI	0.13 (−0.02–0.28)	0.17 (0.01–0.34)	0.20 (0.03–0.36)
AP	0.11 (− 0.02–0.23)	0.14 (0.01–0.27)	0.16 (0.02–0.29)
S	2.85 (0.24–33.50)	3.20 (0.30–34.42)	4.40 (0.15–130.67)

*Abbreviations:* *CD* caesarean delivery; *PA* physical activity; *RERE* relative excess risk due to interaction; *AP* attributable proportion due to interaction and *S* synergy index. RERI> 0, AP > 0, or S > 1 suggest significant additive interaction; *OR* odds ratio; *CI* confidence interval

Model 1: Univariable analyses

Model 2: Multivariable analyses, adjusted for age, body mass index, weight gain from registration to glucose challenge test, gestational age at delivery, habitual smokers before and during pregnancy, alcohol drinkers before and during pregnancy, family history of diabetes in first-degree relatives, parity≥1, Han nationality, systolic blood pressure at registration for pregnancy, birth weight, neonatal height

Model 3: Further adjusted for gestational diabetes, in addition to the variables listed in model 2

### Physical activity and sleep status for risk of caesarean delivery for medical reasons and non-medical reasons

Low PA was associated with increased risk of CD for medical reasons (adjusted OR:1.13, 95%CI: 1.04–1.23) but not associated with CD for non-medical reasons. Sleep duration ≥9 h/day was associated with increased risk of both CD for medical reasons (adjusted OR: 1.12, 95%CI: 1.02–1.22) and CD for non-medical reasons (adjusted OR:1.16, 95%CI: 1.05–1.29). However, sleep duration < 7 h/day and sleep quality were not associated with increased risk of either CD for medical reasons or CD for non-medical reasons (Table 4).

**Table 4:** ORs of Physical activity and sleep status for CD for medical reasons and non-medical reasons

Variables		OR (95% confidence interval)	
		CD for medical reasons	CD for non-medical ressons rereasons
**Model 1**	Physical activity		
	Low V.S Moderate-to-high	1.12 (1.04–1.21)	1.07 (0.97–1.17)
	Sleep duration (h/day)		
	< 7	0.96 (0.71–1.28)	1.40 (1.01–1.95)
	≥7 to < 9	1.00 (Reference)	1.00 (Reference)
	≥9	1.08 (0.99–1.17)	1.11 (1.01–1.23)
	Sleep quality		
	Good	1.00 (Reference)	1.00 (Reference)
	Moderate	0.99 (0.91–1.07)	1.01 (0.91–1.11)
	Poor	0.87 (0.67–1.14)	0.98 (0.72–1.35)
**Model 2**	Physical activity		
	Low V.S Moderate-to-high	1.13 (1.04–1.23)	1.10 (0.99–1.22)
	Sleep duration (h/day)		
	< 7	0.90 (0.65–1.24)	1.37 (0.96–1.94)
	≥7 to < 9	1.00 (Reference)	1.00 (Reference)
	≥9	1.12 (1.02–1.22)	1.16 (1.05–1.29)
	Sleep quality		
	Good	1.00 (Reference)	1.00 (Reference)
	Moderate	0.98 (0.90–1.07)	0.99 (0.89–1.11)
	Poor	0.85 (0.64–1.13)	0.93 (0.66–1.31)
**Model 3**	Physical activity		
	Low V.S Moderate-to-high	1.13(1.04–1.24)	1.11 (0.99–1.23)
	Sleep duration (h/day)		
	< 7	0.88 (0.64–1.22)	1.37 (0.96–1.95)
	≥7 to < 9	1.00 (Reference)	1.00 (Reference)
	≥9	1.12 (1.02–1.22)	1.16 (1.04–1.29)
	Sleep quality		
	Good	1.00 (Reference)	1.00 (Reference)
	Moderate	0.99 (0.91–1.09)	1.01 (0.91–1.13)
	Poor	0.88 (0.66–1.18)	0.98 (0.69–1.38)

*Abbreviations:* *CD* caesarean delivery; *OR* odds ratio

Model 1: Univariable analyses

Model 2: Multivariable analyses, adjusted for age, body mass index, weight gain from registration to glucose challenge test, gestational age at delivery, habitual smokers before and during pregnancy, alcohol drinkers before and during pregnancy, family history of diabetes in first-degree relatives, parity≥1, Han nationality, systolic blood pressure at registration for pregnancy, birth weight, neonatal height

Model 3: Further adjusted for gestational diabetes, in addition to the variables listed in model 2

### Additive interaction between physical activity and sleep duration for risk of caesarean delivery for medical reasons and non-medical reasons

Co-presence of low PA and sleep duration ≥9 h/day was associated with elevated risk of CD for medical reasons (adjusted OR: 1.35, 95%CI: 1.17–1.55) with significant additive interaction (Relative excess risk due to interaction: 0.26, 95%CI: 0.06–0.45; Attributable proportion due to interaction: 0.19, 95%CI: 0.05–0.33). However, their additive interaction for CD for non-medical reasons was not significant (Table 5).

**Table 5:** Additive interaction of physical activity and sleep duration on CD for medical and non-medical reasons

Variables		OR (95% CI)	
		CD for medical reasons	CD for non-medical reasons
**Model 1**	Additive interaction		
	Moderate-to-high PA and sleep time < 9 h/day	1.00(Reference)	1.00(Reference)
	Low PA and sleep time < 9 h/day	1.07(0.96–1.19)	1.07(0.93–1.22)
	Moderate-to-high PA and sleep time ≥ 9/day	1.00(0.89–1.12)	1.08(0.94–1.25)
	Low PA and sleep time ≥ 9 h/day	1.27(1.12–1.44)	1.24(1.05–1.45)
	Additive interaction indications		
	RERI	0.20(0.03–0.37)	0.09(−0.13–0.31)
	AP	0.16(0.03–0.29)	0.07(−0.10–0.25)
	S	3.98(0.26–61.04)	1.60(0.37–6.99)
**Model 2**	Additive interaction		
	Moderate-to-high PA and sleep time < 9 h/day	1.00(Reference)	1.00(Reference)
	Low PA and sleep time < 9 h/day	1.06(0.95–1.20)	1.09(0.95–1.26)
	Moderate-to-high PA and sleep time ≥ 9/day	1.02(0.90–1.15)	1.11(0.95–1.29)
	Low PA and sleep time ≥ 9 h/day	1.34(1.16–1.54)	1.36(1.14–1.61)
	Additive interaction indications		
	RERI	0.26(0.06–0.45)	0.15(−0.09–0.40)
	AP	0.19(0.05–0.33)	0.11(−0.06–0.29)
	S	4.13(0.35–48.48)	1.75(0.54–5.67)
**Model 3**	Additive interaction		
	Moderate-to-high PA and sleep time < 9 h/day	1.00(Reference)	1.00(Reference)
	Low PA and sleep time < 9 h/day	1.07(0.95–1.20)	1.07(0.93–1.24)
	Moderate-to-high PA and sleep time ≥ 9/day	1.01(0.89–1.15)	1.08(0.92–1.26)
	Low PA and sleep time ≥ 9 h/day	1.34(1.16–1.54)	1.36(1.14–1.62)
	Additive interaction indications		
	RERI	0.26(0.06–0.45)	0.21(−0.04–0.45)
	AP	0.19(0.05–0.33)	0.15(−0.02–0.33)
	S	4.21(0.32–56.28)	2.36(0.47–11.79)

*Abbreviations:* *CD* caesarean delivery; *RERE* relative excess risk due to interaction; *AP* attributable proportion due to interaction and S, synergy index. RERI> 0, AP > 0, or S > 1 suggest significant additive interaction; *OR* odds ratio; *CI* confidence interval

Model 1: Univariable analyses

Model 2: Multivariable analyses, adjusted for age, body mass index, weight gain from registration to glucose challenge test, gestational age at delivery, habitual smokers before and during pregnancy, alcohol drinkers before and during pregnancy, family history of diabetes in first-degree relatives, parity ≥1, Han nationality, systolic blood pressure at registration for pregnancy, birth weight, neonatal height

Model 3: Further adjusted for gestational diabetes, in addition to the variables listed in model 2

### Sensitivity analyses

There were statistical differences in some of the variables between the included women and those excluded from the analyses, including age, BMI, weight, SBP, parity, alcohol drinking habit, GDM, weight gain from registration to GCT, sleep duration, gestational age at delivery, birth weight and fetal height (eTable 1). Re-inclusion of the 979 women who had multiple pregnancies or delivered stillbirth did not substantially change the effect sizes of PA and sleep duration for CD (eTable 2). Using moderate-to-high commuting PA as the reference, low commuting PA was associated with CD for non-medical reasons but not with CD for medical reasons. On the contrary, ≤1 h/day housework was associated with increased risk of CD for medical reasons but not CD for non-medical reasons. On the other hand, occupational PA and leisure-time PA were not associated with altered risk of CD for medical reasons and CD for non-medical reasons (eTable 3). More detailed information is available in these tables (see eTables 1, 2, 3).

## Discussion

Our study revealed that (1) low PA and excessive sleep duration during pregnancy were two risk factors for CD; (2) there was a significant synergistic effect of low PA and excessive sleep duration on the risk of CD for medical reasons and overall CD; (3) low PA increased risk of CD for medical reasons while excessive sleep duration increased the risk of both CD for medical reasons and non-medical reasons.

### Implications

Some studies investigated the association between PA and CD but their findings were inconsistent. A prospective longitudinal study with 94 Caucasian pregnant women failed to observe any differences in PA levels during the early second trimester of pregnancy between women with vaginal delivery and those with CD [37]. A secondary analysis of a cluster-randomized trial in Germany (*n* = 1994) showed that lack of PA before or during 12th week of gestation increased risk of CD [38]. A large population-based cohort study with 39,187 Norway nulliparous singleton women found that exercise during pregnancy was associated with a reduced risk of acute CD and high exercise frequency in the 30th week of pregnancy was associated with the greatest risk reduction for elective CD [39]. The differences in period, intensity and types of PA in these studies might be the main reason for these inconsistent findings. In this regard, our study found that low PA increased the risk of CD for medical reasons and overall CD but not CD for non-medical reasons in Chinese pregnant women.

Previous studies also reported inconsistent findings regarding the association of sleep quality and sleep duration with CD. A prospective observational study (*n* = 131) found that poor sleep quality and < 6 h of sleep per day were significantly associated with risk of CD [40]. A meta-analysis of sleep disturbances during pregnancy reported that poor sleep quality and long sleep duration significantly increased risk of CD, while short sleep duration was not associated with CD [41]. A cohort study (*n* = 200) revealed that assisted deliveries, defined as a composite endpoint of CD and instrumental delivery, were more frequent among women with > 8 h/night sleep while CD alone was more frequent among women with < 6.5 h/night sleep [20]. In our study, poor sleep quality and short sleep duration were not significant for CD, possibly due to their low prevalence in our cohort (i.e., 2.3 and 1.9%, respectively). However, our study did find that sleep duration ≥9 h/day increased risk of CD for medical reasons and non-medical reasons. In addition, given to inconsistency in the definition of long sleep duration in previous studies [42], we also performed the same analyses using ≥10 h of sleep per day as excessive sleep duration and found that sleep duration ≥10 h/day had similar effects on CD after adjusting for confounding factors (OR:1.18, 95%CI:1.08–1.29). Excessive sleep duration increased risk of a number of pregnancy complications such as GDM [34], preeclampsia and hypertension [17], late stillbirth [43], and preterm birth [44]. Therefore, avoiding excessive sleep duration might be an important antenatal practice not only to lower the risk of CD but also to reduce other pregnancy complications such as GDM [23].

To our knowledge, there were no studies, to date, reporting interactive effects of PA and sleep duration during pregnancy on the risk of CD. Our study revealed a significant additive interactive effect of low PA and sleep duration ≥9 h/day towards increasing the risk of CD. The additive interaction effect was only significant for CD for medical reasons but not CD for non-medical reasons. Although PA and sleep duration might affect maternal blood glucose metabolism during pregnancy, we did not observe that PA, sleep duration and their interactive effects were mediated via their impacts on GDM. Produced in adipose tissue and placental trophoblasts [45], leptin might be a signaling molecule for placental development and embryonal growth during pregnancy [46]. In early pregnancy, women with physical inactivity or excessive sleep duration were found to have elevated plasma leptin [47, 48]. A prospective longitudinal study found that pregnancies with adverse outcomes had higher a leptin concentration than uncomplicated pregnancies in the first trimester [49]. Therefore, it remained to be tested whether copresence of physical inactivity and excessive sleep duration during pregnancy was related to aberrant placental development, which leaded to high CD risk.

China had the highest CD rate in the world, reaching an overall hospital-based rate of 41.1% in 2016, and the high CD rate was deemed associated with a high medical expense and overuse of medical recourses in China [5]. The CD rate in our cohort was as high as 64%. In this connection, a study in Guangzhou, China, showed that a two-stage intervention program with population health education, skills training for healthcare professionals, equipment and technical support for local healthcare facilities was able to substantially reduce the CD rate, i.e., from 42.4% in 2008 to 35.0% in 2016 [50]. Our study suggested that either to increase PA or/and to maintain recommended sleep duration may reduce about 19% risk of CD for medical reasons due to co-exposure to lack of PA and excessive sleep duration. Such intervention might also have other health benefits such as reduced risk of GDM in the mother and decreased risk of childhood obesity and asthma in the offspring [6]. Indeed, integrated organizational and clinical interventions including health education to pregnant women and professionals are needed to reduce the high CD rate in China, especially in the large cities. PA and sleep should be considered to be included in the intervention package in future intervention studies.

### Strenth and limitations

The major strength of the prospective population-based cohort study was the large sample size, which provided sufficient statistical power to estimate PA, sleep duration and especially for their interactive effect on the risk of CD. Furthermore, our study retrieved the details of the pregnancy outcomes including the indications of CD from the electronic health system, which provided the possibility to explore the specific effect of PA and sleep status on different types of CD. Additionally, many clinical and biochemical risk factors were available to the analyses so that we could adjust for their potential confounding effects.

Our study had several limitations. First, a high proportion of women (34.1%) were not included in the analyses due to missing of key variables such as PA and sleep duration, so the selection bias was not avoidable. However, we had carefully adjusted for potential confounders including but not limited to those who were different between women used in the analyses and those women excluded from the analyses, which may have partially removed their confounding effects if any. Second, body weight at delivery was not systematically documented and not available to the analyses and we could only adjust for weight gain from first care visit to GCT time for the confounding effect of weight gain. The confounding effect of weight gain was only partial removed and the residual confounding could not be completely removed. Also, we cannot exclude the possibility that there were other unmeasured factors that had introduced bias. Third, PA and sleep duration were self-reported and the potential self-reporting bias could not be excluded. Fourth, the majority of the women in the analyses were primipara and had singleton live birth and therefore, our findings could not be extrapolated to multiparous and multiple pregnancy population of women. Fifth, the CD rate of 64% in the central urban areas of Tianjin reported in this study was consistent with the CD rate in the population in 2000 [51] but higher than the overall rate in Chinese supercities, i.e., around 50% and much higher than that in the rural areas [52]. Further validation studies are warranted in other populations, especially in the rural populations.

## Conclusions

The study revealed that low PA and excessive sleep duration during pregnancy increased risk of CD in a synergistic manner, and the significant additive interaction was due to their interactive effect on the risk of CD for medical reasons. Our results highlighted the significance of increasing PA and maintaining recommended sleep duration during pregnancy for perinatal health. Randomized controlled trials were warranted to confirm the effects of promoting PA and maintaining recommended duration of sleep during pregnancy on the rate of CD in Chinese population of pregnant women.

## Supplementary Information

**Additional file 1 **Supplemental tables of sensitivity analyses. Illustrates the details of sensitivity analyses, including **eTable S1**, **eTable S2** and **eTable S3**.

## Data Availability

The datasets used and analysed during the current study are available from the corresponding author on reasonable request.

## Abbreviations

CD: Caesarean delivery
PA: Physical activity
BMI: Body mass index
SBP: Systolic blood pressure
DBP: Diastolic blood pressure
GDM: Gestational diabetes mellitus
GCT: Glucose challenge test
SD: Standard deviation
OR: Odds ratio
CI: Confidence interval
