# Supplementary material for: Physical activity and sleep duration during pregnancy have interactive effects on caesarean delivery: a population-based cohort study in Tianjin, China
Source: BMC Pregnancy Childbirth. 2021 May 28;21:406. doi: 10.1186/s12884-021-03788-4 (PMC8161996; doi:10.1186/s12884-021-03788-4)
Supplement: Supplementary file 1 — Additional file 1 Supplemental tables of sensitivity analyses. Illustrates the details of sensitivity analyses, including eTable S1, eTable S2 and eTable S3. [file 12884_2021_3788_MOESM1_ESM.pdf]

Supplemental tables

eTable 1. Sensitivity analysis of characteristics in included subjects and excluded subjects

Variables	Included	Excluded	P-value
n	13015	9287	
**Variables at registration for pregnancy**			
Age, year	28.48 ± 2.92	28.64 ± 3.07	<0.001[*]
Pre-pregnancy BMI, kg/m^2	22.32 ± 3.38	22.47 ± 3.57	0.002[*]
Han-nationality	12401 (95.3%)	8901 (95.8%)	0.050[**]
Weight, kg	59.53 ± 9.73	59.83 ± 10.22	0.026[*]
Height, cm	163.22 ± 4.72	163.11 ± 4.69	0.081[*]
Systolic blood pressure, mmHg	105.72 ± 10.74	105.42 ± 10.63	0.044[*]
Diastolic blood pressure, mmHg	68.52 ± 7.78	68.34 ± 7.73	0.090[*]
Parity ≥1	484 (3.7%)	422 (4.5%)	0.002[**]
Family history of diabetes in first degree relatives	1125 (9.2%)	710 (8.7%)	0.237[**]
Gestational age at registration, weeks	10.38 ± 2.33	10.58 ± 2.65	<0.001[*]
History of caesarean delivery ≥1	262 (2.0%)	162 (1.74%)	0.079[**]
**Variables at Glucose challenge test**			
Smoke before or during pregnancy	665 (5.1%)	443 (4.8%)	0.263[**]
Drink before or during pregnancy	4126 (31.7%)	2628 (28.3%)	<0.001[**]
Gestational diabetes mellitus	897 (7.3%)	642 (8.2%)	0.024[**]
Glucose challenge test, mmol/L	6.63 ± 1.56	6.63 ± 1.66	0.916[*]
Weight gain from registration to GCT, kg	7.64 ± 3.17	6.98 ± 4.27	<0.001[*]
Occupational physical activity			<0.001[**]
Low	11257 (86.5%)	2365 (94.8%)	
Moderate	1741 (13.4%)	129 (5.2%)	
High	9 (0.1%)	2 (0.1%)	
Commuting physical activity(min/day) [a]			<0.001[**]
low	12273 (94.4%)	2405 (97.4%)	
Moderate-to-high	725 (5.6%)	64 (2.6%)	
Leisure-time physical activity(min/week)			<0.001[*]
0	7025(54.0%)	1499(64.3%)	
<150	3764 (28.9%)	624 (26.8%)	
≥150	2173 (16.8%)	205 (8.8%)	
Housework physical activity(h/day)			0.7240[**]
≤1	9983 (78.6%)	1069 (79.1%)	
>1	2716 (21.4%)	283 (20.9%)	
Physical activity during pregnancy [b]			
Low	7027 (54.0%)	676 (55.2%)	0.441[**]

Moderate-to-high	5988 (46.0%)	549 (44.8%)	
Sleep duration during pregnancy(h/day)			0.005**
<7	251 (1.9%)	35 (1.8%)	
≥7 to <9	5568 (42.8%)	768 (39.0%)	
≥9	7196 (55.3%)	1166 (59.2%)	
Sleep quality during pregnancy			0.1050**
Good	4913 (37.9%)	999 (39.8%)	
Moderate	7765 (59.8%)	1460 (58.2%)	
Poor	299 (2.3%)	48 (1.9%)	
**Variables at delivery**			
Gestational age at delivery, weeks	39.08 ± 1.50	37.32 ± 6.25	<0.001*
Birth weight, kg	3.38 ± 0.47	3.36 ± 0.48	0.001*
Neonatal height, cm	50.15 ± 1.64	50.05 ± 1.71	<0.001*

Abbreviations: BMI, body mass index; GCT, glucose challenge test; CD, caesarean delivery.

*Derived from Student's t-test; **Derived from Chi-square Test or Fisher's exact test.

a Low was defined was defined barely outgoing, using motorized transportation or <30 min/day walking or cycling to and from work; b Low was defined when subjects simultaneously reported the light level of occupational physical activity, low commuting physical activity, <150min/week of leisure-time physical activity, and ≤1 h/day housework physical activity; and others were defined as 'moderate-to-high'

eTable 2. Sensitivity analysis of reclusion of the 979 subjects who had multiple pregnancies or delivered stillbirth

Variables	OR (95% confidence interval)		
	Model 1	Model 2	Model 3
Physical activity			
Low	1.11 (1.03-1.19)	1.13 (1.04-1.22)	1.13 (1.04-1.22)
Moderate-to-high	1.00 (Reference)	1.00 (Reference)	1.00 (Reference)
Sleep duration (h/day)			
<7	1.11 (0.85-1.44)	1.06 (0.79-1.41)	1.04 (0.78-1.40)
≥7 to <9	1.00 (Reference)	1.00 (Reference)	1.00 (Reference)
≥9	1.10 (1.02-1.18)	1.14 (1.05-1.23)	1.14 (1.05-1.23)
Sleep quality			
Good	1.00 (Reference)	1.00 (Reference)	1.00 (Reference)
Moderate	1.01 (0.94-1.08)	1.00 (0.92-1.08)	1.01 (0.93-1.10)
Poor	0.90 (0.71-1.15)	0.87 (0.67-1.14)	0.90 (0.69-1.18)

Abbreviations: OR, odds ratio.

Model 1: Univariable analysis.

Model 2: Multivariable analysis, adjusted for age, body mass index, weight gain from registration to glucose challenge test, gestational age at delivery, habitual smokers before and during pregnancy, alcohol drinkers before and during pregnancy, family history of diabetes in first-degree relatives, parity≥1, Han nationality, systolic blood pressure at registration for pregnancy, birth weight, neonatal height.

Model 3: Further adjusted for gestational diabetes, in addition to the variables listed in model 2.

eTable 3. Sensitivity analysis of commuting, housework, occupational and leisure-time PAs on CD for medical reasons or non-medical reasons

Variables		OR (95% confidence interval)	
		CD for medical reasons	CD for non-medical reasons
**Model 1**	Commuting physical activity [a]		
	Low V.S Moderate-to-high	1.23 (1.04-1.45)	1.42 (1.14-1.76)
	Housework physical activity(h/day)		
	≤1 V.S >1	1.15 (1.04-1.26)	1.00 (0.89-1.12)
	Occupational physical activity		
	Low V.S Moderate-to-high	1.09 (0.97.1.22)	1.06 (0.93-1.22)
	Leisure-time physical activity(min/week)		
	<150 V.S ≥150	1.04 (0.94-1.16)	1.02 (0.90-1.16)
**Model 2**	Commuting physical activity [a]		
	Low V.S Moderate-to-high	1.15 (0.96-1.37)	1.38 (1.10-1.75)
	Housework physical activity(h/day)		
	≤1 V.S >1	1.22 (1.10-1.35)	1.05 (0.93-1.19)
	Occupational physical activity		
	Low V.S Moderate-to-high	1.09 (0.96-1.23)	1.07 (0.92-1.24)
	Leisure-time physical activity(min/week)		
	<150 V.S ≥150	1.05 (0.94-1.18)	1.06 (0.92-1.21)
**Model 3**	Commuting physical activity [a]		
	Low V.S Moderate-to-high	1.18 (0.99-1.42)	1.42 (1.12-1.80)
	Housework physical activity(h/day)		
	≤1 V.S >1	1.22 (1.10-1.36)	1.06 (0.94-1.21)
	Occupational physical activity		
	Low V.S Moderate-to-high	1.09 (0.96-1.23)	1.08 (0.93-1.26)
	Leisure-time physical activity(min/week)		
	<150 V.S ≥150	1.04 (0.93-1.17)	1.06 (0.92-1.21)

Abbreviations: CD, caesarean delivery, OR, odds ratio, PA, physical activity.

a Low was defined was defined barely outgoing, using motorized transportation or <30 min/day walking or cycling to and from work.

Model 1: Univariable analysis.

Model 2: Multivariable analysis, adjusted for age, body mass index, weight gain from registration to glucose challenge test, gestational age at delivery, habitual smokers before and during pregnancy, alcohol drinkers before and during pregnancy, family history of diabetes in first-degree relatives, parity≥1, Han nationality, systolic blood pressure at registration for pregnancy, birth weight, neonatal height.

Model 3: Further adjusted for gestational diabetes, in addition to the variables listed in model 2.
